# FOXD1 promotes EMT and cell stemness of oral squamous cell carcinoma by transcriptional activation of SNAI2

**DOI:** 10.1186/s13578-021-00671-9

**Published:** 2021-08-04

**Authors:** Yang Chen, Weilian Liang, Ke Liu, Zhengjun Shang

**Affiliations:** 1 The State Key Laboratory Breeding Base of Basic Science of Stomatology, Hubei Province and Key Laboratory of Oral Biomedicine (Wuhan University), Ministry of Education (Hubei-MOST KLOS & KLOBM), Wuhan, China; 2 Department of Oral and Maxillofacial-Head and Neck Oncology, School and Hospital of Stomatology, Wuhan University, 237 Luoyu Road, Hongshan District, Wuhan, 430079 China

**Keywords:** FOXD1, EMT, STEMNESS, SNAI2, OSCC

## Abstract

**Background:**

Epithelial-mesenchymal transition (EMT) and cell stemness are implicated in the initiation and progression of oral squamous cell carcinoma (OSCC). Revealing the intrinsic regulatory mechanism may provide effective therapeutic targets for OSCC.

**Results:**

In this study, we found that Forkhead box D1 (FOXD1) was upregulated in OSCC compared with normal samples. Patients with a higher FOXD1 expression had a poorer overall survival and disease-free survival. Immunohistochemical staining results showed that FOXD1 expression was related to the clinical stage and relapse status of OSCC patients. When FOXD1 expression was knocked down in CAL27 and SCC25 cells, the migration, invasion, colony formation, sphere formation, and proliferation abilities decreased. Moreover, EMT and stemness-related markers changed remarkably, which indicated that the EMT process and cell stemness were inhibited. Conversely, overexpression of FOXD1 promoted EMT and cell stemness. Further study demonstrated that FOXD1 could bind to the promoter region and activate the transcription of SNAI2. In turn, the elevated SNAI2 affected EMT and cell stemness. An in vivo study showed that FOXD1-overexpressing CAL27 cells possessed a stronger tumorigenic ability.

**Conclusions:**

Our findings revealed a novel mechanism in regulating EMT and cell stemness and proposed FOXD1 as a potential marker for the diagnosis and treatment of OSCC.

**Supplementary Information:**

The online version contains supplementary material available at 10.1186/s13578-021-00671-9.

## Background

Oral squamous cell carcinoma (OSCC) is the most common cancer of head and neck squamous cell carcinoma (HNSCC). HNSCC ranks as the 7th most common cancer worldwide, and over 430,000 deaths related to HNSCC are reported annually [1, 2]. Despite dramatic advances in diagnosis and therapy strategies, the prognosis of OSCC remains poor owing to the high recurrence and metastasis rate [3]. Therefore, finding key genes and regulatory pathways controlling the progression of OSCC is especially imperative.

Forkhead box D1 (FOXD1), a member of the Forkhead family, was first identified in the forebrain neuroepithelium and has been demonstrated to be a vital gene participating in the development of the kidney and retina [4]. Previous studies have shown that FOXD1 also participates in the development of various cancers, including liver cancer [5], cervical cancer [6], pancreatic cancer [7], breast cancer [8], and glioma [9]. For instance, Sun et al. found that lncRNA NORAD promotes cell stemness and angiogenesis in liver cancer by regulating the miR-211-5p/FOXD1/VEGF-A axis [5]; Cheng et al. found that FOXD1 can determine the renewal ability and tumorigenicity of glioma through transcriptional regulation of ALDH1A3 [9]. Recently, FOXD1 was found to be significantly highly expressed in OSCC tissues and related to overall survival, disease-free survival, and metastasis status [10]. Nevertheless, the function of FOXD1 in OSCC remains unclear.

Epithelial-mesenchymal transition (EMT) is a process during which epithelial tumor cells lose their polarity and cell–cell adhesions and then transform into a mesenchymal cell phenotype. Cancer cells that have undergone EMT display lower E-cadherin and higher N-cadherin and vimentin expression and possess stronger migration and invasion abilities [11]. Recent studies have demonstrated that the EMT process is associated with cell stemness in various cancers. For example, Pastushenko et al. revealed that the initiation, progression, invasiveness, metastasis, and stemness of squamous cell carcinoma are promoted in a hybrid EMT state, which is induced by the functional loss of FAT1 [12]. Our previous study also demonstrated that the interaction between CCL21/CCR7 can regulate EMT and cell stemness [13]. Tumor cells with enhanced stemness possess stronger self-renewal ability and tumorigenicity [14]. However, whether FOXD1 participates in regulating EMT and stemness in OSCC remains unknown at present.

In this study, we found that FOXD1 is upregulated in OSCC and correlated with poor clinical outcomes. Then, we demonstrated that FOXD1 can promote EMT and cell stemness in OSCC. Further study showed that FOXD1 promotes the transcriptional activity of SNAI2, which is a key regulatory gene related to EMT and cell stemness. This study reveals the role and mechanism of FOXD1 in regulating tumor progression and proposes FOXD1 as a novel therapeutic target for OSCC.

## Materials and methods

### Specimen collection

A total of 60 OSCC and 8 normal oral mucosa specimens were collected from the Hospital of Stomatology, Wuhan University. Our research was permitted by the Ethics Committee of Wuhan University (IRB-ID: 2021A18). Written informed consent was obtained from each participant. The clinicopathological characteristics of the patients are available in Additional file 1: Table S1.

### Cell lines and culture

The OSCC cell lines CAL27, SCC25, and HN4 were cultivated with culture medium containing 10% fetal bovine serum (FBS, Natocor, Córdoba, Argentina). The cell lines mentioned above were obtained from the China Center for Type Culture Collection (Shanghai, China). Human immortalized oral epithelial cells (HIOECs) were generously donated by Professor Chengzhang Li and cultivated in KGM™ Gold Keratinocyte Cell Basal Medium (Lonza, Walkersville, MD) with the associated supporting growth factors. All cells were cultured at 37 °C in humid conditions with 5% CO_2_.

### Cell transfection

Short hairpin RNAs (shRNAs; GeneChem, Shanghai, China) were used to knockdown FOXD1 expression (Additional file 1: Table S2). Recombinant lentiviruses (GeneChem) were used to obtain stable FOXD1 overexpression or knockdown cell lines. To identify stable FOXD1 overexpression or knockdown cell lines, the transfected cells were cultured with 2 μg/mL puromycin for 7 days. Small interfering RNAs (siRNAs; Hanbio, Shanghai, China) were used to silence SNAI2 expression (Additional file 1: Table S3). Lipofectamine™ 3000 (Invitrogen, Carlsbad, CA) was used to transfect siRNAs and shRNAs.

### Real-time PCR (RT-PCR)

We conducted these experiments as described previously [13]. The primers that we employed in this research are shown in Additional file 1: Table S3; (Additional file 1: Table S4).

### Western blot

The experiment was carried out following our previously published protocol [13]. Anti-FOXD1 antibody was purchased from Genetex (1:1000; CA, USA). Anti-E-cadherin, anti-N-cadherin, and anti-vimentin antibodies were purchased from Cell Signaling Technology (1:1000; MA, USA); anti-CD44, anti-ALDH1A1, anti-BMI1, and anti-SNAI2 antibodies were purchased from Proteintech (1:1000; Wuhan, China). The gray value of the band was analyzed with ImageJ software (National Institutes of Health, Bethesda, MD, USA).

### Wound healing and Matrigel invasion assays

The experiments were performed as previously described [13]. In brief, a scratch was made after the cells grew into a confluent monolayer. Then, the bottom of the 6-well plate was marked with a black marker to confirm that the images were taken at the same place 0 and 24 h postwounding. The migrated areas were assessed by Image-Pro Plus 6.0 (migrated area = wound area at 0 h–wound area at 24 h). Five different fields were calculated in the wound healing assay. Serum-free medium was used to inhibit cell proliferation.

For the Matrigel invasion assay, 50 μl Matrigel (BD Biosciences, San Jose, CA) was used to precoat the Transwell chamber. Then, 2 × 10^5^ cells were seeded in the upper chamber. Forty‐eight hours later, the invading cells were stained and counted.

### Transwell migration assay

In brief, 2 × 10^5^ cells were pretreated with 10 μg/ml mitomycin C (MedChemExpress, NJ, USA) for 2 h, resuspended in FBS-free medium and seeded on the upper layer of the chamber. Twenty-four hours later, the migrated cells on the lower layer of the chamber were stained and counted.

### Sphere forming and colony formation assays

Four hundred cells were seeded into each well of 12-well plates and then cultured for 10 days to observe colony formation ability. Then, the colonies were fixed and stained with crystal violet. Colonies were photographed and counted under a light microscope.

The 6-well culture plates were pretreated with polyHEMA as previously described to prepare low adhesion dishes [13]. Cells were seeded into the pretreated plate (1000 cells/well) and cultivated with standard cancer stem cell medium. After 12 days of cultivation, the quality of spheres was observed under a microscope.

### CCK-8 assay

The experiment was conducted as previously described [13]. Briefly, cells were seeded into 96-well plates and incubated with a mixture of 100 μl medium and 10 μl Cell Counting Kit‐8 (CCK‐8, Biosharp, Hefei, China) for 24, 48, and 72 h. Subsequently, the proliferation ability of the cells was determined by the optical density (OD) at 450 nm.

### EdU incorporation assay

EdU detection was performed using the BeyoClick™ EdU Cell Proliferation Kit with Alexa Fluor 594 (Beyotime, Shanghai, China). OSCC cells (1 × 10^4^) were evenly seeded into each well of 24-well culture plates and then cultivated with the EdU reagents. Two hours later, the cells were fixed with 4% paraformaldehyde (Servicebio, Wuhan, China). Then, cell staining was performed following the manufacturer’s explanatory memorandum. Finally, images were obtained with a fluorescence microscope (Biozero BZ-8000, Keyence, Osaka, Japan).

### Luciferase reporter assay

The pGL4-basic plasmid containing the SNAI2 promoter, the pGL4-basic luciferase plasmid, and the phRL-TK plasmid were purchased from Miaolingbio (Wuhan, China). The pGL4-basic plasmid containing the SNAI2 promoter and the phRL-TK plasmids were cotransfected into cells with Lipofectamine™ 3000. Cells transfected with the phRL-TK plasmid and pGL4-basic luciferase plasmid were used as negative controls. Forty-eight hours later, luciferase activity was determined by the Luciferase Assay System Kit (Promega, USA) according to the manufacturer’s instructions.

### Immunohistochemistry (IHC) staining

Paraffin‐embedded OSCC tissues, normal mucosae, and xenograft tumor sections underwent IHC staining using anti-FOXD1 (1:100), anti-E-cadherin (1:400), anti-CD44 (1:200), and anti-SNAI2 (1:400) antibodies. The IHC staining process was carried out as described in our previous study [13]. Image-Pro Plus 6.0 was used to assess the area and the integrated optical density (IOD) value of the section stained by IHC. The mean densitometry of the image (magnification, ×400) was designated as the representative staining intensity.

### Mouse xenografts

All animal experiments were approved by the Ethics Committee of the Hospital of Stomatology at Wuhan University. Twenty-four female BALB/c nude mice were purchased from Beijing Vital River Laboratory Animal Technology Co., Ltd. (Beijing, China). CAL27 cells transfected with a FOXD1 overexpression or knockdown lentivirus were used for this assay. The mice were randomly divided into four groups (n = 6), and 3 × 10^6^ CAL27 cells were injected subcutaneously into the mice. Thirty-five days later, the tumors were removed and analyzed.

## Results

### FOXD1 is upregulated in OSCC and correlated with poor clinical outcomes

**Fig. 1:**
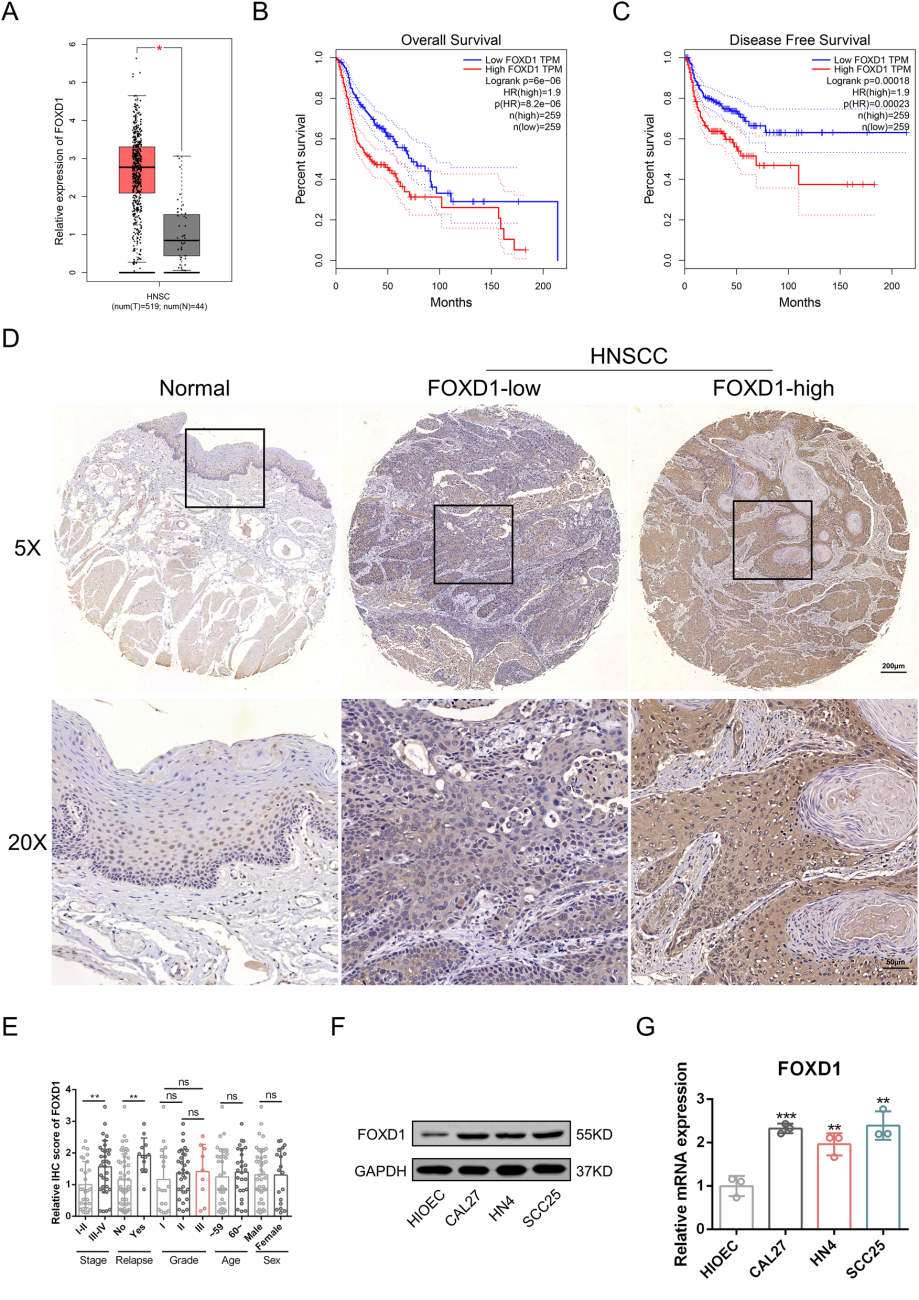
FOXD1 is upregulated in OSCC and correlated with poor clinical outcomes. **A** The expression of FOXD1 in HNSCC and normal samples acquired from GEPIA. **B** Overall survival rates according to FOXD1 expression in HNSCC. **C** Disease-free survival rates according to FOXD1 expression in HNSCC. **D** Representative IHC images of FOXD1 in clinical specimens. **E** FOXD1 expression in OSCC of different clinical stages, relapse status, grades, ages and genders. n = 60. **F** The protein expression of FOXD1 in different cell lines. **G** Relative mRNA expression of FOXD1 in different cell lines

First, FOXD1 expression in OSCC was assessed. TCGA data from the Gene Expression Profiling Interactive Analysis (GEPIA) web tool [15] showed that the expression of FOXD1 in OSCC samples was significantly higher than that in normal samples (Fig. 1A). Additionally, the overall survival and disease-free survival analysis acquired from GEPIA demonstrated that patients with higher FOXD1 expression had a worse prognosis (Fig. 1B, C). Next, FOXD1 expression in 60 OSCC tissues and 8 normal mucosae samples was detected using IHC staining (Fig. 1D). We found that the expression of FOXD1 was related to the clinical stage and relapse status but not to the sex, age, or pathological grade of OSCC patients (Fig. 1E). Western blot and PCR results revealed that the OSCC cell lines CAL27, SCC25, and HN4 possessed a higher FOXD1 expression than the normal oral epithelial cell line HIOEC (Fig. 1F, G). In general, the abovementioned results indicated that FOXD1 was upregulated in OSCC and predicted poor clinical outcomes.

### FOXD1 knockdown inhibits EMT of OSCC

**Fig. 2:**
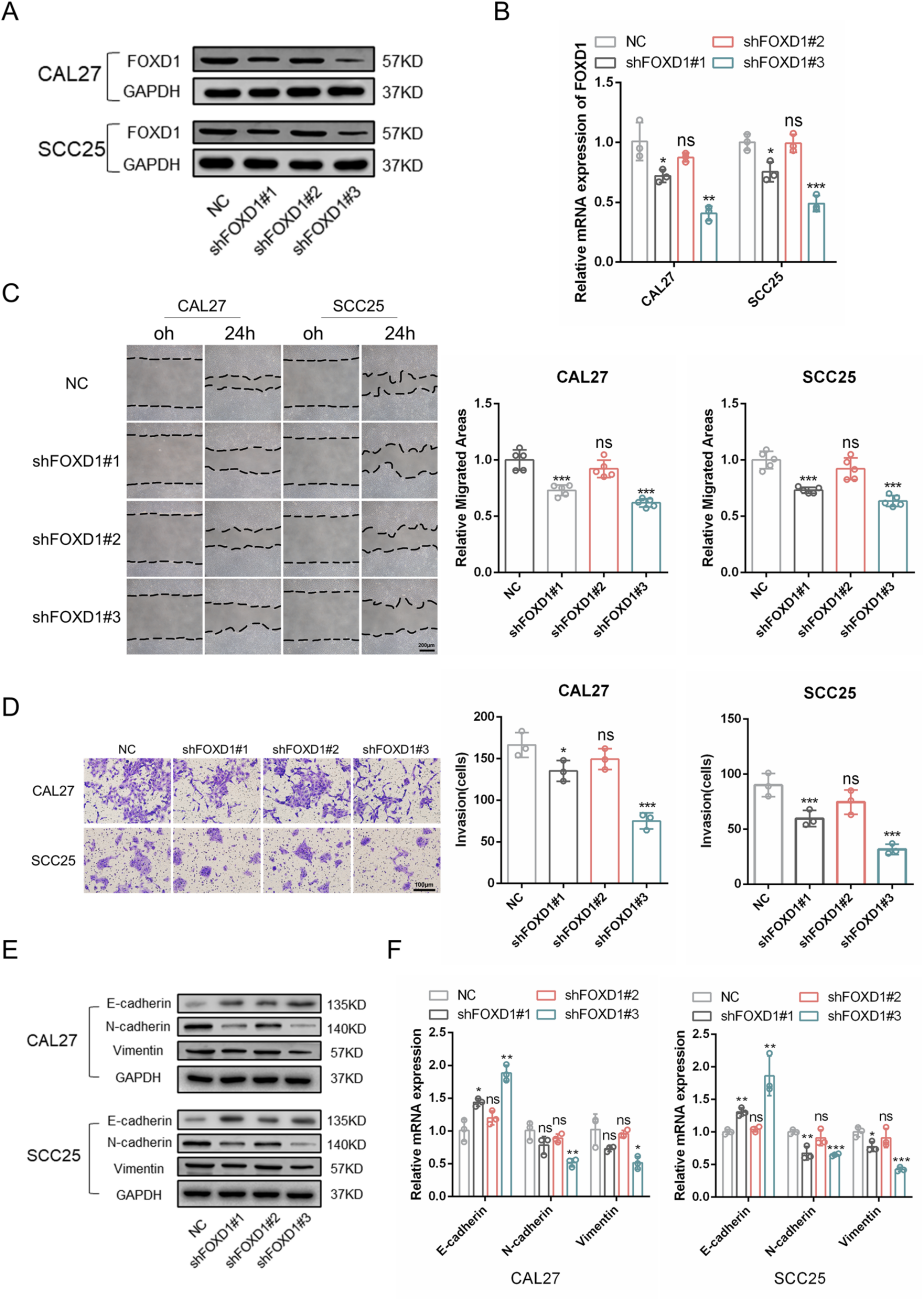
FOXD1 knockdown inhibits EMT of OSCC. **A** The protein expression of FOXD1 in OSCC cells transduced with FOXD1 shRNAs. **B** Relative mRNA expression of FOXD1 in OSCC cells transfected with FOXD1 shRNAs. **C** The migration areas of OSCC cells transduced with FOXD1 shRNAs. **D** The invasion results of OSCC cells transduced with FOXD1 shRNAs. **E** The protein expression of EMT-related markers in OSCC cells transduced with FOXD1 shRNAs. **F** Relative mRNA expression of EMT-related markers in OSCC cells transduced with FOXD1 shRNAs

To reveal the role and intrinsic regulatory mechanism of FOXD1 in OSCC progression, we knocked down FOXD1 expression in CAL27 and SCC25 cells using three different shRNAs. Forty-eight hours after transfection, western blotting and PCR were used to detect FOXD1 expression. We found that shFOXD1#3 was the most effective sequence for interfering with FOXD1 expression in both CAL27 and SCC25 cells (Fig. 2A, B). Then, wound healing, Transwell migration and Matrigel invasion assays were performed. We found that the migration and invasion abilities were decreased after FOXD1 knockdown (Fig. 2C, D; Additional file 2: Fig. S2A). EMT is a process closely correlated with the occurrence and development of OSCC [16]. Here, we found that knockdown of FOXD1 significantly promoted E-cadherin expression and reduced N-cadherin and vimentin expression, which implies an inhibited EMT status in the tumor cells (Fig. 2E, F). Collectively, these results demonstrated that silencing FOXD1 inhibited the migration, invasion, and EMT of OSCC.

### FOXD1 knockdown decreases cell stemness of OSCC

**Fig. 3:**
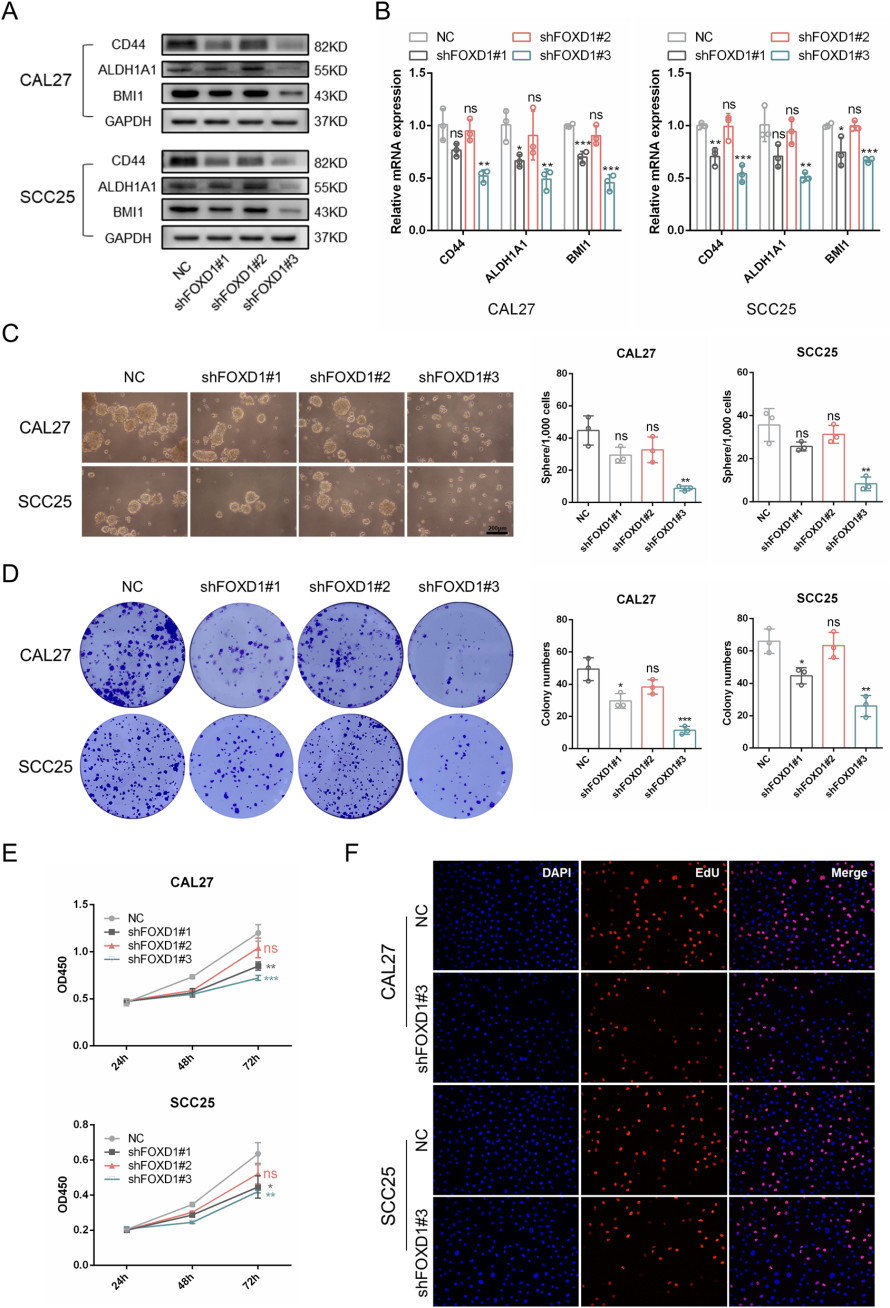
FOXD1 knockdown decreases cell stemness of OSCC. **A** The protein expression of CSCs-related markers in OSCC cells transduced with FOXD1 shRNAs. **B** Relative mRNA expression of CSCs-related markers in OSCC cells transduced with FOXD1 shRNAs. **C** The sphere formation results of OSCC cells transduced with FOXD1 shRNAs. **D** The colony formation results of OSCC cells transduced with FOXD1 shRNAs. **E** The proliferation ability of OSCC cells transduced with FOXD1 shRNAs. **F** The proliferative cells of OSCC cells transduced with FOXD1 shRNAs

In our previous study, we demonstrated that the EMT process of tumor cells was closely related to their cell stemness [13]. Here, we found that the CSC-related markers CD44, ALDH1A1, and BMI1 were decreased at the protein and mRNA levels after FOXD1 knockdown (Fig. 3A, B). In addition, colony formation and sphere formation assays indicated that FOXD1 knockdown markedly impaired the self-renewal ability of OSCC cells (Fig. 3C, D). In addition, the CCK-8 assay revealed a lower proliferation ability of FOXD1 knockdown cells (Fig. 3E). The EdU assay showed fewer proliferative CAL27 and SCC25 cells when FOXD1 was inhibited (Fig. 3F). Altogether, the findings verified that FOXD1 knockdown decreased the stemness of OSCC.

### FOXD1 overexpression promotes EMT and cancer stem-like properties of OSCC

**Fig. 4:**
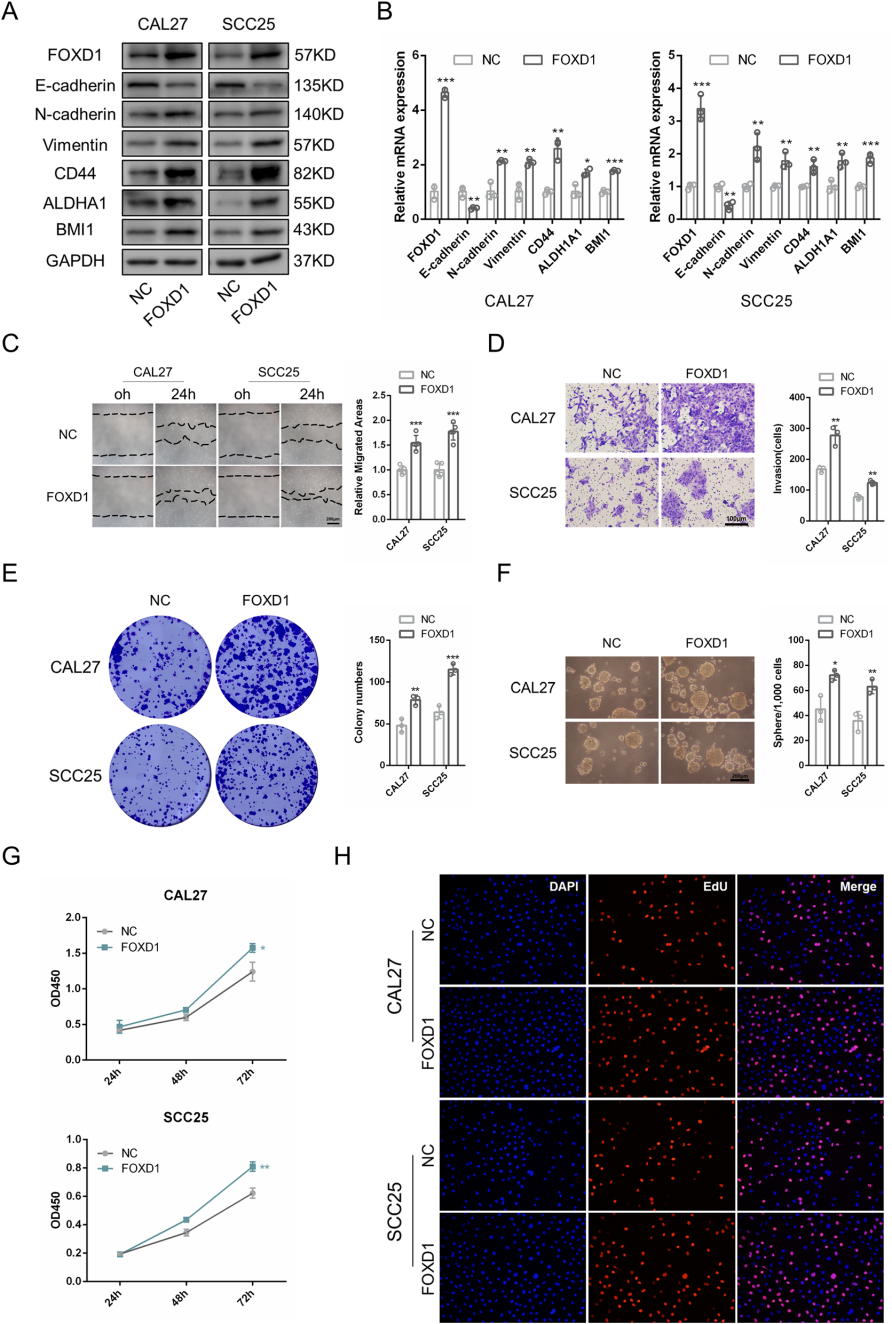
FOXD1 overexpression promotes EMT and cancer stem-like properties of OSCC. **A** The protein level of EMT and CSCs related markers in FOXD1 overexpression OSCC cells. **B** Relative mRNA level of EMT and CSCs related markers in FOXD1 overexpression OSCC cells. **C** The migration areas of FOXD1 overexpression OSCC cells. **D** The invasion results of FOXD1 overexpression OSCC cells. **E** The colony formation results of FOXD1 overexpression OSCC cells. **F** The sphere formation results of FOXD1 overexpression OSCC cells. **G** The proliferation ability of FOXD1 overexpression OSCC cells. **F** The proliferative cells of FOXD1 overexpression OSCC cells

We overexpressed FOXD1 in CAL27 and SCC25 cells using a lentivirus transduction system. Western blotting and PCR were used to detect FOXD1 expression in transfected cells after they were treated with 2 μg/ml puromycin for 1 week (Fig. 4A, B). In contrast to FOXD1 knockdown, FOXD1 overexpression dramatically reduced E-cadherin expression and increased N-cadherin, vimentin, CD44, ALDH1A1, and BMI1 expression at both the protein and RNA levels (Fig. 4A, B). In addition, the areas of migration were larger, and the numbers of migrated and invaded cells were larger (Fig. 4C, D; Additional file 2: Fig. S1B). The colony formation and sphere formation abilities were stronger (Fig. 4E, F). CCK-8 and EdU assays indicated that FOXD1 overexpression increased the proportion of proliferative cells and promoted cell proliferation (Fig. 4G, H). Jointly, FOXD1 overexpression promoted EMT and cell stemness of OSCC cells.

### SNAI2 is a target of FOXD1

**Fig. 5:**
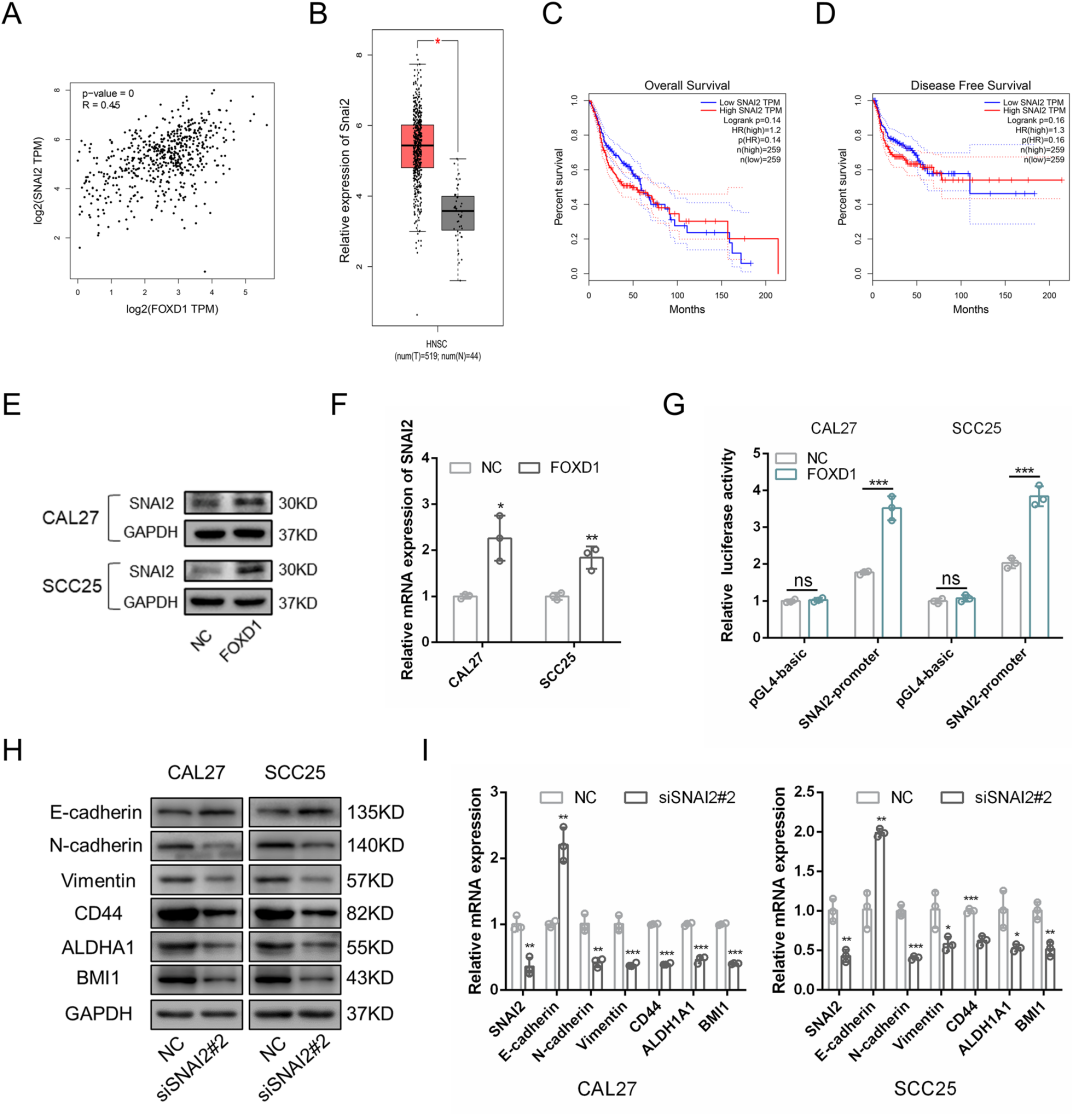
SNAI2 is a target of FOXD1. **A** The correlation analysis of FOXD1 and SNAI2 acquired from GEPIA. **B** The expression of SNAI2 in HNSCC and normal samples acquired from GEPIA. **C** Overall survival rates according to SNAI2 expression in HNSCC. **D** Disease-free survival rates according to SNAI2 expression in HNSCC. **E** Relative protein expression of SNAI2 in FOXD1 overexpression OSCC cells. **F** Relative mRNA expression of SNAI2 in FOXD1 overexpression OSCC cells. **G** Relative transcriptional activity of SNAI2 in FOXD1 overexpression OSCC cells. **H** The protein expression of EMT and CSCs related markers in SNAI2 knockdown OSCC cells. **I** Relative mRNA expression of EMT and CSCs related markers in SNAI2 knockdown OSCC cells

SNAI1, SNAI2, TWIST1, TWIST2, ZEB1, ZEB2, NANOG, SOX2, and POU5F1 are the key regulatory genes contributing to the EMT process and cell stemness [17, 18]. The correlation analysis acquired from GEPIA showed that SNAI2 expression was positively correlated with FOXD1 expression (Fig. 5A), while other genes were not (Additional file 2: Fig. S1A), indicating that SNAI2 might be a target of FOXD1. Then, SNAI2 expression in FOXD1 knockdown and overexpression cells was assessed separately. Consistent with the data obtained from GEPIA, we found that SNAI2 expression was positively correlated with FOXD1 expression in both CAL27 and SCC25 cells (Fig. 5E, F; Additional file 2: Fig. S1B, C). FOXD1 is a transcription factor that plays a role by regulating the transcription of its target genes. The results acquired from JASPAR [19] showed that there were several binding sites for FOXD1 in the promoter region of SNAI2 (Additional file 1: Table S5). To verify whether FOXD1 could directly regulate the transcriptional activity of SNAI2, a dual-luciferase reporter assay was performed. The results showed markedly increased luciferase activity in FOXD1-overexpressing cells, indicating that FOXD1 could promote the transcription of SNAI2 (Fig. 5G). Next, we examined the role of SNAI2 in CAL27 and SCC25 cells. Three specific siRNAs were used to interfere with the expression of SNAI2. The results confirmed that siSANI2#2 was the most effective sequence for knocking down SNAI2 expression (Additional file 2: Fig. S1D). In addition, we found that SNAI2 silencing substantially inhibited the EMT process and reduced cell stemness (Fig. 5H, I), which was similar to the results caused by FOXD1 knockdown. Additionally, we found that SNAI2 was highly expressed in OSCC samples compared with normal samples (Fig. 5B). Nonetheless, the expression of SNAI2 was not correlated with clinical prognosis (Fig. 5C, D). Collectively, these results revealed that SNAI2 was a target for FOXD1. FOXD1 promoted EMT and enhanced cell stemness by transcriptional activation of SNAI2.

### FOXD1 promotes OSCC progression in vivo

**Fig. 6:**
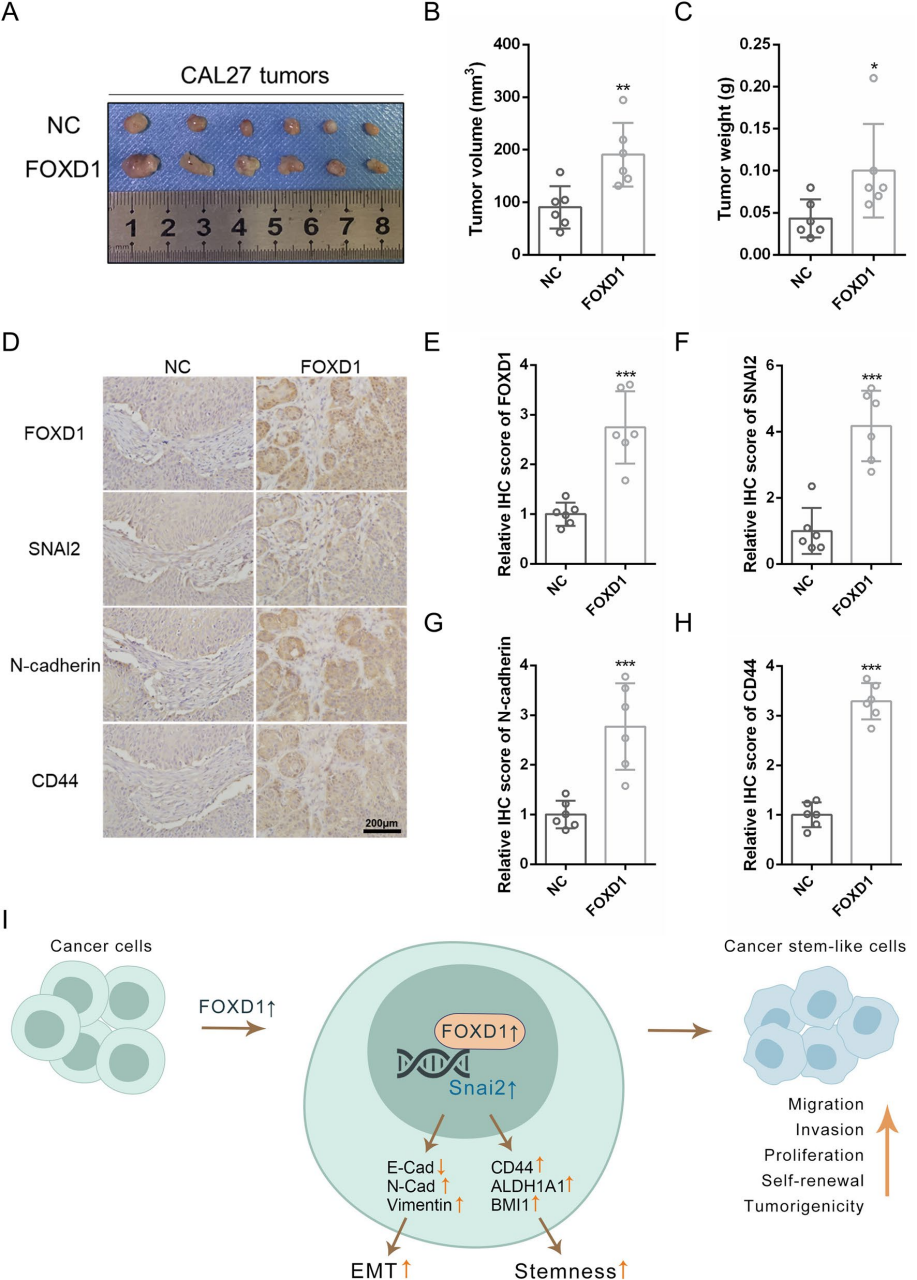
FOXD1 promotes OSCC progression in vivo. **A** Xenograft tumors of negative control (NC) and FOXD1 groups. **B** Tumor volume NC of and FOXD1 groups. **C** Tumor weight of NC and FOXD1 groups. **D** Representative IHC images of FOXD1, SNAI2, E‐cadherin, and CD44 in NC and FOXD1 groups in xenograft tumors. **E** Relative IHC score of FOXD1. **F** Relative IHC score of SNAI2. **G** Relative IHC score of N‐cadherin. **H** Relative IHC score of CD44. **I** Schematic diagram of the roles of FOXD1 in OSCC

To further investigate the role of FOXD1 in tumorigenicity, a mouse xenograft model was used. FOXD1 overexpression and knockdown CAL27 cells were prepared in vitro. Then, the cells were subcutaneously injected into the mice separately. On the 45^th^ day, we dissected and assessed the tumors. As expected, FOXD1-overexpressing cells possessed obviously stronger tumor formation ability (Fig. 6A). Both the volume and weight of tumors in the FOXD1 overexpression group were higher than those in the negative control group (Fig. 6B, C). In contrast, the tumor formation ability of FOXD1 knockdown cells was greatly suppressed (Additional file 2: Fig. S2C). The volume and weight of tumors in the FOXD1 knockdown group were also lower than those in the negative control group (Additional file 2: Fig. S2D, E). In addition, tumors of the FOXD1 overexpression group displayed increased expression of FOXD1, SNAI2, N-cadherin and CD44 (Fig. 6D–H). Overall, the results demonstrated that FOXD1 promoted OSCC progression in vivo by regulating EMT and cell stemness.

## Discussion

Surgery, chemotherapy, and radiotherapy are traditional therapeutic strategies for OSCC. However, due to their existing limitations, the 5-year survival rate of OSCC is less than 50% [3, 20]. Therefore, finding genes that are crucial for tumor progression may provide new therapeutic targets for OSCC. Recent studies have demonstrated that upregulated FOXD1 is related to the metastasis status and adverse clinical outcomes of OSCC [10]. In addition, FOXD1-AS1 can enhance the proliferation and decrease the apoptosis of nasopharyngeal carcinoma by upregulating FOXD1 expression [21]. These results indicate that FOXD1 can behave as an oncogene in cancer. In our research, FOXD1 was upregulated in OSCC and related to poor clinical outcomes. Silencing FOXD1 inhibited the EMT process and decreased cell stemness. Conversely, overexpression of FOXD1 promoted EMT and cell stemness. Notably, FOXD1-overexpressing cells showed stronger tumorigenic ability. In addition, we verified that SNAI2, which is closely related to EMT and cell stemness, was the target of FOXD1. FOXD1 could bind to the promoter region and activate the transcription of SNAI2 (Fig. 6I).

EMT is a process during which cancer cells lose their polarity and convert to a spindle‐like mesenchymal morphology [11]. Parikh et al. found that a partial EMT population in oral cancer is closely correlated with lymph node metastasis, perineural invasion, and tumor grade [22]. Zhang et al. revealed that CD100 motivates the EMT process, thus leading to a higher metastasis possibility of HNSCC [23]. EMT plays an important role in cancer initiation, progression, recurrence, and metastasis [24]. Here, we found that upregulated FOXD1 was related to an adverse prognosis in OSCC. Knockdown of FOXD1 significantly decreased the migration and invasion abilities and inhibited the EMT process of OSCC. In contrast, overexpression of FOXD1 promoted EMT and increased migration and invasion abilities. Very recently, Chen et al. found that FOXD1 can affect chemoresistance and EMT by regulating lncRNA CYTOR in OSCC [25]. Our results are consistent with their studies, indicating that FOXD1 can affect the prognosis of OSCC by regulating the EMT process.

Cancer stem cells, a minority cluster of cells in tumors, possess the abilities of self-renewal and initiating tumor formation from very few cells [26]. Leticia et al. found that treatment with JQ1 can reduce stemness and result in less invasive and more chemosensitive breast cancer [27]. Muhammad et al. revealed that c-Fos overexpression promotes EMT, cell stemness, and tumor growth when compared with control cells [28]. Cancer cells with increased stemness have stronger self-renewal and tumorigenesis abilities and contribute to adverse clinical outcomes [29]. Previous studies illustrated that FOXD1 can regulate the stemness of mesenchymal glioma stem cells [9]. However, the relationship between FOXD1 and the stemness of OSCC cells is currently unclear. Here, we found that cell stemness was decreased when FOXD1 was silenced in OSCC. Conversely, overexpression of FOXD1 enhanced self-renewal, proliferation, and tumorigenesis. Our findings indicated that FOXD1 is involved in the progression of OSCC by regulating cell stemness.

Snai2, a transcription factor closely related to EMT and stemness [17, 30], was positively correlated with the expression of FOXD1 in our research. Fan et al. found that SNAI2 can induce EMT by suppressing the transcriptional activity of miR-222-3p and upregulating the expression of PDCD10 [31]. Tian et al. found that SNAI2 promotes the stemness of prostate cancer cells potentially by modulating the GSK-3β/β-catenin pathway [32]. Here, we found that FOXD1 bound to the promoter region of SNAI2 and promoted its transcription. Silencing SANI2 inhibited EMT and decreased cell stemness. For the first time, we identified SNAI2 as the downstream target gene of FOXD1. We revealed that FOXD1 could promote EMT and cell stemness in OSCC by transcriptional activation of SNAI2. Interestingly, although SNAI2 was the target for FOXD1, SNAI2 itself was not a good prognostic marker for OSCC.

Li et al. found that upregulated FOXD1 is related to the metastasis status and adverse clinical outcomes of OSCC [10]. However, they did not reveal the intrinsic mechanism of FOXD1 in regulating tumor progression. Wang et al. demonstrated that FOXD1-AS1 can regulate glycolysis in nasopharyngeal carcinoma by sustaining FOXD1 expression, thus promoting tumor progression [21]. Our previous studies have demonstrated that EMT and stemness are closely related to OSCC progression [13, 33]. However, whether FOXD1 affects the progression of OSCC by regulating EMT and stemness is unclear at present. In this study, we verified the roles of FOXD1 in regulating EMT and cell stemness. For the first time, we identified SNAI2 as the downstream target gene of FOXD1. As EMT and cell stemness are closely related to tumor occurrence, recurrence, and metastasis, our studies link FOXD1 to the current understanding of OSCC pathogenesis.

## Conclusion

We found that FOXD1 is upregulated in OSCC and correlated with adverse clinical outcomes. We verified that FOXD1 can promote EMT and cell stemness by transcriptional activation of SNAI2. Our studies expand the current understanding of FOXD1 in tumor biology and provide a promising molecular target for the diagnosis and treatment of OSCC.

## Supplementary Information

**Additional file 1: Table S1.** The clinical pathological characteristics of patients. **Table S2.** Targeting sequences for shRNAs. **Table S3.** Targeting sequences for siRNAs. **Table S4.** Sequences of PCR primers. **Table S5.** The potential binding sites of FOXD1 in SNAI2 promoter region.

**Additional file 2: Fig. S1.** (A) The correlation analysis between FOXD1 and the key regulatory genes contributing to the EMT process and cell stemness. (B) The protein expression of SNAI2 in OSCC transduced with different FOXD1 shRNAs. (C) Relative mRNA expression of SNAI2 in OSCC cells transduced with shFOXD1#3. (D) Relative protein expression of SNAI2 in OSCC transduced with different SNAI2 shRNAs. **Fig. S2.** (A) The number of migrated cells of OSCC cells transduced with FOXD1 shRNAs. (B) The number of migrated cells of FOXD1 overexpression OSCC cells. (C) Xenograft tumors of NC and FOXD1 knockdown groups. (D) Tumor volume NC of and FOXD1 knockdown groups. (E) Tumor weight of NC and FOXD1 knockdown groups.

**Additional file 3:** The densitometric data of western blot images.

## Data Availability

The datasets used and analysed during the current study are available from the corresponding author on reasonable request.

## Abbreviations

EMT: Epithelial-mesenchymal transition
HNSCC: Head and neck squamous cell carcinoma
OSCC: Oral squamous cell carcinoma
FOXD1: Forkhead box D1
shRNAs: Short hairpin RNAs
siRNAs: Small interference RNAs
IHC: Immunohistochemistry
GEPIA: Gene Expression Profiling Interactive Analysis
