# Supplementary material for: FOXD1 promotes EMT and cell stemness of oral squamous cell carcinoma by transcriptional activation of SNAI2
Source: Cell Biosci. 2021 Aug 4;11:154. doi: 10.1186/s13578-021-00671-9 (PMC8335989; doi:10.1186/s13578-021-00671-9)
Supplement: Supplementary file 1 — Additional file 1: Table S1. The clinical pathological characteristics of patients. Table S2. Targeting sequences for shRNAs. Table S3. Targeting sequences for siRNAs. Table S4. Sequences of PCR primers. Table S5. The potential binding sites of FOXD1 in SNAI2 promoter region. [file 13578_2021_671_MOESM1_ESM.docx]

**Table S1. Clinical pathological characteristics of patients**

| ***Patients*** | ***Type*** | ***Sex*** | ***Age*** | ***Location*** | ***TNM*** | ***Relapse*** | ***Grade*** |
| --- | --- | --- | --- | --- | --- | --- | --- |
| 1 | OSCC | Male | 43 | Tongue | T1N0M0 | No | II |
| 2 | OSCC | Male | 87 | Palate | T1N0M0 | No | I-II |
| 3 | OSCC | Female | 60 | Tongue | T1N0M0 | Yes | II |
| 4 | OSCC | Male | 61 | Gingiva | T1N0M0 | No | II |
| 5 | OSCC | Male | 50 | Tongue | T1N0M0 | No | II |
| 6 | OSCC | Male | 52 | Tongue | T1N0M0 | No | I |
| 7 | OSCC | Male | 53 | Buccal mucosa | T2N0M0 | No | I |
| 8 | OSCC | Male | 45 | Palate | T2N0M0 | Yes | I |
| 9 | OSCC | Male | 72 | Buccal mucosa | T2N0M0 | Yes | II |
| 10 | OSCC | Female | 57 | Tongue | T2N0M0 | No | II |
| 11 | OSCC | Female | 66 | Tongue | T2N0M0 | No | II |
| 12 | OSCC | Female | 52 | Tongue | T2N0M0 | No | III |
| 13 | OSCC | Male | 53 | Mouth floor | T2N0M0 | Yes | I |
| 14 | OSCC | Male | 56 | Tongue and pharyngeal | T2N0M0 | No | II |
| 15 | OSCC | Female | 80 | Buccal mucosa | T2N0M0 | No | I |
| 16 | OSCC | Female | 80 | Gingiva | T2N0M0 | No | II |
| 17 | OSCC | Male | 64 | Tongue | T2N0M0 | No | II |
| 18 | OSCC | Male | 68 | Tongue | T2N0M0 | No | II-III |
| 19 | OSCC | Female | 70 | Gingiva | T2N0M0 | Yes | II-III |
| 20 | OSCC | Male | 56 | Gingiva | T2N0M0 | No | II |
| 21 | OSCC | Female | 55 | Lip | T2N0M0 | No | II |
| 22 | OSCC | Male | 69 | Soft plate | T2N0M0 | Yes | II |
| 23 | OSCC | Male | 36 | Tongue | T2N0M0 | No | I |
| 24 | OSCC | Female | 58 | Gingiva | T2N0M0 | Yes | II-III |
| 25 | OSCC | Male | 63 | Tongue | T2N0M0 | No | I-II |
| 26 | OSCC | Female | 34 | Tongue | T2N0M0 | No | I-II |
| 27 | OSCC | Male | 55 | Tongue | T2N1M0 | Yes | II-III |
| 28 | OSCC | Male | 52 | Tongue | T3N0M0 | No | II |
| 29 | OSCC | Male | 59 | Tongue | T3N1M0 | No | I-II |
| 30 | OSCC | Male | 71 | Soft plate | T2N1M0 | Yes | II |
| 31 | OSCC | Male | 53 | Tongue | T2N1M0 | No | I-II |
| 32 | OSCC | Male | 53 | Lip | T2N1M0 | No | I-II |
| 33 | OSCC | Male | 56 | Tongue and gingiva | T2N0M0 | No | II |
| 34 | OSCC | Male | 53 | Tongue and mouth floor | T3N1M0 | No | II |
| 35 | OSCC | Female | 54 | Tongue | T3N1M0 | No | I-II |
| 36 | OSCC | Female | 48 | Tongue | T2N1M0 | Yes | II |
| 37 | OSCC | Male | 47 | Tongue | T3N0M0 | No | II |
| 38 | OSCC | Female | 71 | Buccal mucosa | T2N1M0 | No | II |
| 39 | OSCC | Male | 55 | Tongue and pharyngeal | T2N1M0 | No | II |
| 40 | OSCC | Female | 55 | Gingiva | T3N0M0 | No | I |
| 41 | OSCC | Male | 66 | Mouth floor | T4N2bM0 | No | II |
| 42 | OSCC | Male | 47 | Tongue | T2N2M0 | No | II |
| 43 | OSCC | Male | 81 | Tongue | T2N2bM0 | No | II |
| 44 | OSCC | Male | 51 | Tongue and mouth floor | T4N2cM0 | No | II |
| 45 | OSCC | Female | 41 | Buccal mucosa | T4N0M0 | Yes | I-II |
| 46 | OSCC | Female | 67 | Tongue | T2N2M0 | No | I-II |
| 47 | OSCC | Female | 68 | Buccal mucosa | T2N2M0 | No | II |
| 48 | OSCC | Male | 80 | Gingiva | T4N0M0 | No | II |
| 49 | OSCC | Male | 60 | Buccal mucosa, gingiva, plate and mouth floor | T4N2bM0 | No | II-III |
| 50 | OSCC | Female | 77 | Buccal mucosa and gingiva | T4N2M0 | No | II-III |
| 51 | OSCC | Male | 60 | Buccal mucosa | T1N2M0 | Yes | I-II |
| 52 | OSCC | Male | 71 | Soft plate | T4N0M0 | No | III |
| 53 | OSCC | Male | 44 | Tongue | T4N2M0 | No | II |
| 54 | OSCC | Male | 45 | Plate | T4N0M0 | No | II-III |
| 55 | OSCC | Male | 60 | Buccal mucosa | T3N2bM0 | No | II |
| 56 | OSCC | Male | 56 | Mouth floor | T4N1M0 | No | I-II |
| 57 | OSCC | Male | 62 | Mouth floor | T4N1M0 | No | II |
| 58 | OSCC | Male | 77 | Buccal mucosa | T4N1M0 | No | II |
| 59 | OSCC | Male | 76 | Mouth floor and tongue | T2N2cM0 | No | II |
| 60 | OSCC | Female | 43 | Tongue | T1N0M0 | No | I |
| 61 | Normal mucosa | Male | 62 | Buccal |  |  |  |
| 62 | Normal mucosa | Male | 49 | Plate |  |  |  |
| 63 | Normal mucosa | Female | 85 | Tongue |  |  |  |
| 64 | Normal mucosa | Female | 59 | Tongue |  |  |  |
| 65 | Normal mucosa | Male | 63 | Tongue |  |  |  |
| 66 | Normal mucosa | Male | 62 | Gingiva |  |  |  |
| 67 | Normal mucosa | Male | 76 | Tongue |  |  |  |
| 68 | Normal mucosa | Male | 77 | Gingiva |  |  |  |

**Table S2. Targeting sequences for shRNAs**

| **Gene** | **ID** | **Targeting sequences (5′ to 3′)** |
| --- | --- | --- |
| FOXD1 | shRNA#1 | TGTCCAGTGTCGAGAACTTTA |
|  | shRNA#2 | GCCCTTCTCCATCGAGAGCAT |
|  | shRNA#3 | CCTCGCCGAGGAAACAGACAT |

**Table S3. Targeting sequences for siRNAs**

| **Gene** | **ID** | **Sense (5′ to 3′)** | **Antisense (3′ to 5′)** |
| --- | --- | --- | --- |
| SNAI2 | siRNA#1 | CCGGAUACUCCUCAUCUUUTT | AAAGAUGAGGAGUAUCCGGTT |
|  | siRNA#2 | GCAUUUGCAGACAGGUCAATT | UUGACCUGUCUGCAAAUGCTT |
|  | siRNA#3 | GCUCAUCUGCAGACCCAUUTT | AAUGGGUCUGCAGAUGAGCTT |

**Table S4. Sequences of PCR primers**

| **Genes** | **Targeting sequences (5′ to 3′)** |
| --- | --- |
| FOXD1 | Forward: TATATCGCGCTCATCACTATGG |
|  | Reverse: CTGATGAACTCACAGATCTCGC |
| E-cadherin | Forward: GCCTCCTGAAAAGAGAGTGGAAG |
|  | Reverse: TGGCAGTGTCTCTCCAAATCCG |
| N-cadherin | Forward: CGATAAGGATCAACCCCATACA |
|  | Reverse: TTCAAAGTCGATTGGTTTGACC |
| Vimentin | Forward: TGAATGACCGCTTCGCCAACTAC |
|  | Reverse: CTCCCGCATCTCCTCCTCGTAG |
| CD44 | Forward: TCTGAATCAGATGGACACTCAC |
|  | Reverse: CATTGCCACTGTTGATCACTAG |
| ALDH1A1 | Forward: GACAATGCTGTTGAATTTGCAC |
|  | Reverse: AAGGATATACTTCTTAGCCCGC |
| BMI1 | Forward: CAAGACCAGACCACTACTGAAT |
|  | Reverse: TATCTTCATCTGCAACCTCTCC |
| SNAI2 | Forward: CTGTGACAAGGAATATGTGAGC |
|  | Reverse: CTAATGTGTCCTTGAAGCAACC |
| GAPDH | Forward: GGAGCGAGATCCCTCCAAAAT |
|  | Reverse: GGCTGTTGTCATACTTCTCATGG |

**Table S5. The potential binding sites of FOXD1 in SNAI2 promoter region**

| Matrix ID | Name | Relative score | Start | End | Strand | Predicted sequence |
| --- | --- | --- | --- | --- | --- | --- |
| MA0031.1 | FOXD1 | 0.851736315 | 630 | 637 | - | GAAAACAA |
| MA0031.1 | FOXD1 | 0.843740049 | 1804 | 1811 | + | TTAAACAC |
| MA0031.1 | FOXD1 | 0.821148909 | 1891 | 1898 | - | GTGAACAG |
| MA0031.1 | FOXD1 | 0.805293005 | 256 | 263 | - | GAAAACAC |
