# Supplementary material for: FOXD1 promotes EMT and cell stemness of oral squamous cell carcinoma by transcriptional activation of SNAI2
Source: Cell Biosci. 2021 Aug 4;11:154. doi: 10.1186/s13578-021-00671-9 (PMC8335989; doi:10.1186/s13578-021-00671-9)
Supplement: Supplementary file 2 — Additional file 2: Fig. S1. (A) The correlation analysis between FOXD1 and the key regulatory genes contributing to the EMT process and cell stemness. (B) The protein expression of SNAI2 in OSCC transduced with different FOXD1 shRNAs. (C) Relative mRNA expression of SNAI2 in OSCC cells transduced with shFOXD1#3. (D) Relative protein expression of SNAI2 in OSCC transduced with different SNAI2 shRNAs. Fig. S2. (A) The number of migrated cells of OSCC cells transduced with FOXD1 shRNAs. (B) The number of migrated cells of FOXD1 overexpression OSCC cells. (C) Xenograft tumors of NC and FOXD1 knockdown groups. (D) Tumor volume NC of and FOXD1 knockdown groups. (E) Tumor weight of NC and FOXD1 knockdown groups. [file 13578_2021_671_MOESM2_ESM.docx]

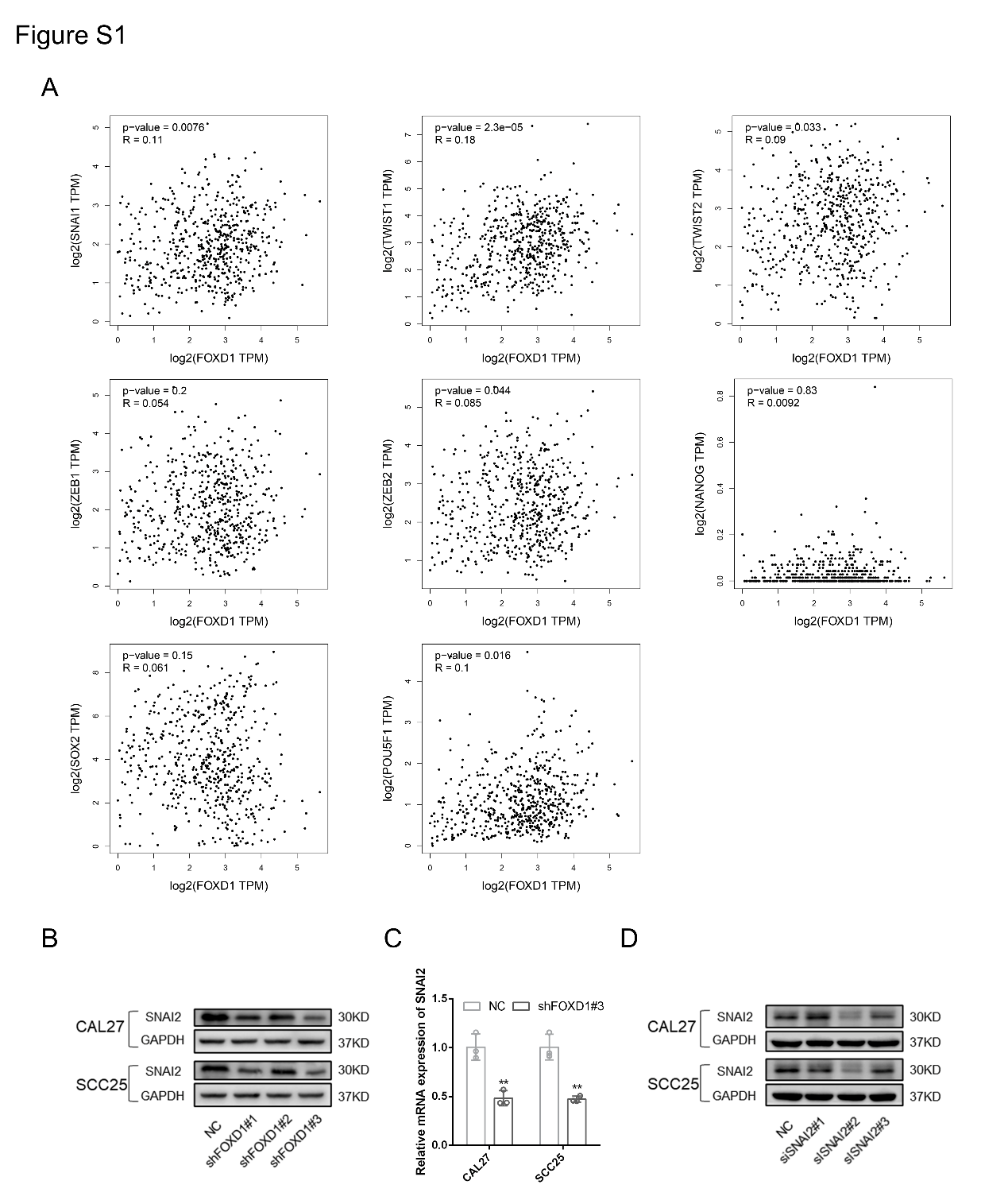


**Fig. S1.** (A) The correlation analysis between FOXD1 and the key regulatory genes contributing to the EMT process and cell stemness. (B) The protein expression of SNAI2 in OSCC transduced with different FOXD1 shRNAs. (C) Relative mRNA expression of SNAI2 in OSCC cells transduced with shFOXD1#3. (D) Relative protein expression of SNAI2 in OSCC transduced with different SNAI2 shRNAs.


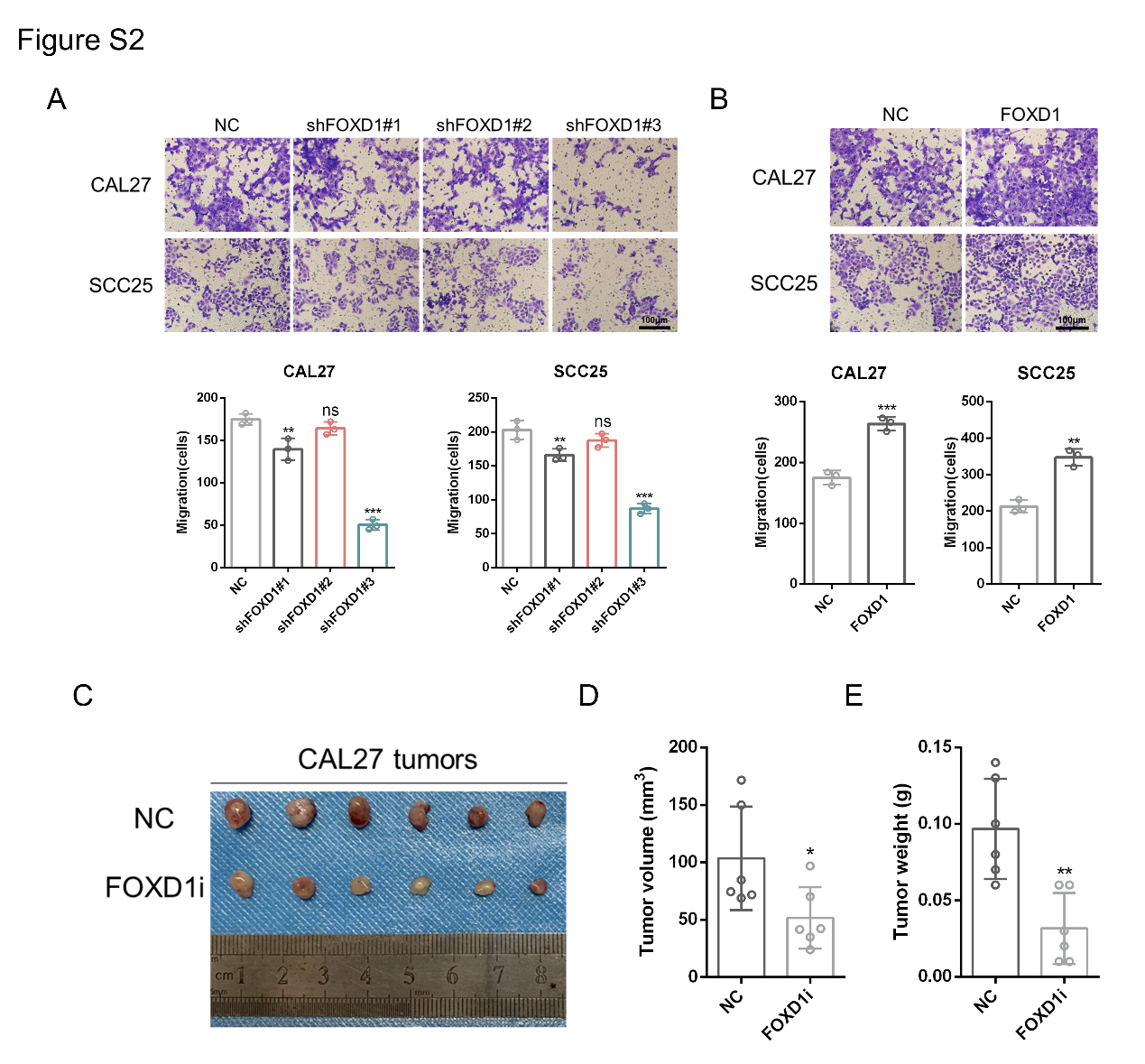


**Fig. S2.** (A) The Number of migrated cells of OSCC cells transduced with FOXD1 shRNAs. (B) The Number of migrated cells of FOXD1 overexpression OSCC cells. (C) Xenograft tumors of NC and FOXD1 knockdown groups. (D) Tumor volume NC of and FOXD1 knockdown groups. (E) Tumor weight of NC and FOXD1 knockdown groups.
